# Control of Axonal Growth and Regeneration of Sensory Neurons by the p110δ PI 3-Kinase

**DOI:** 10.1371/journal.pone.0000869

**Published:** 2007-09-12

**Authors:** Britta J. Eickholt, Aminul I. Ahmed, Meirion Davies, Evangelia A. Papakonstanti, Wayne Pearce, Michelle L. Starkey, Antonio Bilancio, Anna C. Need, Andrew J. H. Smith, Susan M. Hall, Frank P. Hamers, Karl P. Giese, Elizabeth J. Bradbury, Bart Vanhaesebroeck

**Affiliations:** 1 Medical Research Council Centre for Developmental Neurobiology, King's College London, London, United Kingdom; 2 Neurorestoration Group, Wolfson Centre for Age-Related Diseases, King's College London, London, United Kingdom; 3 Ludwig Institute for Cancer Research, London, United Kingdom; 4 Centre for the Cellular Basis of Behaviour, Institute of Psychiatry, King's College London, London, United Kingdom; 5 Gene Targeting Laboratory, The Institute for Stem Cell Research, University of Edinburgh, Edinburgh, United Kingdom; 6 Rudolf Magnus Institute of Neuroscience, University Medical Centre Utrecht, Utrecht, The Netherlands; 7 Department of Biochemistry and Molecular Biology, University College London, London, United Kingdom; Columbia University, United States of America

## Abstract

The expression and function of the 8 distinct catalytic isoforms of PI 3-kinase (PI3K) in the nervous system are unknown. Whereas most PI3Ks have a broad tissue distribution, the tyrosine kinase-linked p110δ isoform has previously been shown to be enriched in leukocytes. Here we report that p110δ is also highly expressed in the nervous system. Inactivation of p110δ in mice did not affect gross neuronal development but led to an increased vulnerability of dorsal root ganglia neurons to exhibit growth cone collapse and decreases in axonal extension. Loss of p110δ activity also dampened axonal regeneration following peripheral nerve injury in adult mice and impaired functional recovery of locomotion. p110δ inactivation resulted in reduced neuronal signaling through the Akt protein kinase, and increased activity of the small GTPase RhoA. Pharmacological inhibition of ROCK, a downstream effector of RhoA, restored axonal extension defects in neurons with inactive p110δ, suggesting a key role of RhoA in p110δ signaling in neurons. Our data identify p110δ as an important signaling component for efficient axonal elongation in the developing and regenerating nervous system.

## Introduction

Phosphoinositide 3-kinases (PI3Ks) are a family of lipid kinases which regulate a wide variety of biological responses in different cell types [1]. In the nervous system, PI3K activity contributes to the establishment of appropriate connectivity by regulating various cellular processes, including neuronal differentiation, survival, migration, extension and guidance [2]–[5]. The 8 isoforms of mammalian PI3Ks have been grouped into three classes (I, II, and III) [1]. The class IA subset of PI3Ks signal downstream of Tyr kinases and Ras, and are heterodimers composed of one of three p110 catalytic subunits-p110α, p110β or p110δ-in complex with one of the three regulatory subunit (collectively called ‘p85s’). Detailed information on the tissue distribution of the p110α and p110β isoforms is not available, although evidence for a broad expression of both isoforms has been presented [6]–[9]. On the other hand, p110δ is known to be highly enriched in leukocytes [7], [10], [11]. Gene-targeting studies in the mouse have uncovered non-redundant roles of specific p110 PI3K isoforms in immunity, metabolism and cardiac function [12], [13]. In contrast, the expression and function of the distinct PI3K isoforms in the nervous system have not been investigated.

Here, we report that expression of p110δ PI3K is highly enriched in the embryonic nervous system in the mouse at stages concomitant with the extension and guidance of neuronal processes. Genetic or pharmacological inactivation of p110δ in sensory neurons led to a reduction in PI3K signaling, increased sensitivity to growth cone collapse and deficient axonal elongation under limiting growth conditions. In addition, mice with inactive p110δ show impaired axonal regeneration and functional recovery following a sciatic nerve crush injury. These results identify p110δ-mediated PI3K signaling as a crucial component for efficient axonal elongation.

## Results and Discussion

### p110δ expression is highly enriched in the nervous system

**Figure 1:**
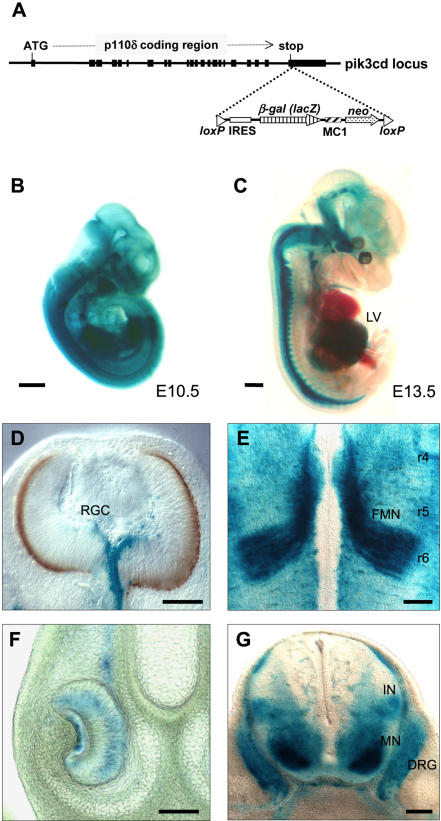
Expression pattern of p110α and p110δ as assessed by X-gal staining of lacZ (β-Gal) reporter mice. (A) p110δ gene locus of p110δ ^lz^ mice. (B) Broad expression of p110α/LacZ in p110α ^lz^ mice at E10.5. Scale bar, 500 µm (C) Side view of an E13.5 p110δ ^lz^ embryo reveals high X-gal staining in the developing central and peripheral nervous system, and the liver (LV). Scale bar, 1 mm. (D) p110δ/LacZ expression in retinal ganglion cells (RGC) in the central retina at E12.5. Scale bar, 100 µm (E) p110δ/LacZ expression in the facial motor nuclei (FMN) within the hindbrain as they migrate from rhombomere (r) 4 to their final position in r6 at E12.5. Scale bar, 150 µm. (F) p110δ/LacZ expression in axonal processes and cell bodies within the vomeronasal epithelium (shown here at E15.5). Scale bar, 100 µm. (G) p110δ/LacZ expression in the DRG, motor neuron pool (MN) and interneurons (IN) in cross-sections through the spinal cord at E13.5. Scale bar, 100 µm.

To date, no detailed information on the distribution of the distinct PI3K isoforms in neuronal tissue has been reported. In order to analyze the expression of p110α and p110δ, reporter mice were generated in which a *β*-Gal/*LacZ* reporter gene was inserted into the endogenous *p110α* or *p110δ* gene locus by homologous recombination [14], [15]. An internal ribosome entry site (IRES)-*LacZ* sequence was targeted into the last exon of the p110 gene, immediately after the stop codon (schematically shown for p110δ in Figure 1A). This allowed independent production of the p110 protein and the β-Gal enzyme from the same bicistronic mRNA, encoded by the p110 gene promoter(s). These mice are hereafter referred to as *p110α ^lz^* and *p110δ ^lz^* mice.

As expected, the p110α isoform of PI3K was found to be widely expressed (Figure 1B), and high expression of p110δ in the embryonic liver, the principal location of hemopoiesis, supports previous reports on the expression of p110δ in adult mice leukocytes [7], [11]. Unexpectedly strong signals of p110δ were also observed in the nervous system, especially in the spinal cord, dorsal root ganglia (DRG), cranial sensory ganglia and peripheral nerves (Figure 1C). Stainings at various time points showed that this pattern of *LacZ* expression generally reflects the appearance of differentiated neurons in both the central and peripheral nervous system. At E12.5, for example, the *p110δ/*LacZ signal followed the wave of retinal ganglion cell differentiation in the central retina (Figure 1D), and was not detected in the neuroblast layer at any developmental stage analyzed (E12–E18; Figure 1D and data not shown). In addition, *p110δ/*LacZ expression was enriched during neuronal migration, for example at E12.5 in the facial motor nuclei within the hindbrain during movement to the final rhombomere location (Figure 1E). Axonal processes and cell bodies within the vomeronasal epithelium also expressed *p110δ/*LacZ, shown in Figure 1F. Cross sections through the spinal cord at lumbar levels revealed highly enriched *p110δ/*LacZ staining in the DRG, interneurons of the spinal cord and the spinal motor neuron pool (Figure 1G).

In adult mice, high *p110δ/*LacZ expression was also present in neurons, for example in specific brain regions, including the hippocampus, cortex and thalamus (Figure S1A–C). Immunoblotting of brain extracts confirmed the enrichment of p110δ protein in distinct brain regions (Figure S1D). This contrasts with the uniform distribution of p110α and p110β, as well as various forms of the p85 regulatory subunits (Figure S1D).

### Inactivation of p110δ increases the vulnerability of sensory neurons to growth cone collapse and decreases axonal extension

To determine the contribution of p110δ to neuronal development and function, we analyzed mice in which p110δ was inactivated as a result of the introduction of a germline point mutation which renders the kinase inactive (p110δ^D910A^; [14]. Homozygous p110δ^D910A/D910A^ mice, hereafter referred to as p110δ kinase-inactive (KI) mice, are viable and fertile [14]. In these mice, expression of the mutated p110δ protein and the other PI3K subunits was equivalent to that of the wild-type (WT) proteins in brain homogenate (Figure S2), demonstrating the absence of compensatory PI3K expression. Gross morphology of the nervous system (data not shown) and hippocampus-dependent learning behavior (Figure S3) were unaffected in p110δ KI mice, suggesting that the establishment and functioning of the neuronal circuitry required for complex behavioral tasks does not depend on p110δ activity.

**Figure 2:**
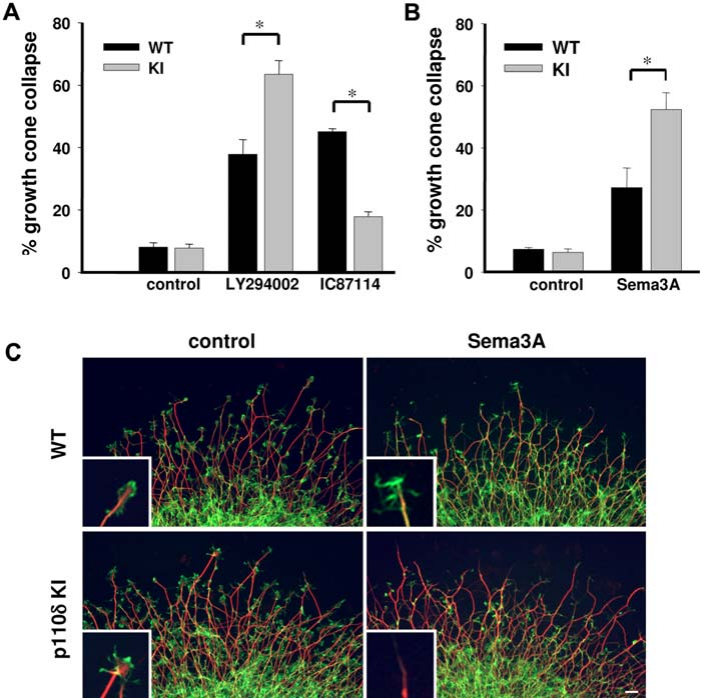
Increased sensitivity of p110δ KI DRG growth cones to collapse in response to PI3K inhibition or Sema3A treatment. (A) Growth cone collapse in DRG explants induced by LY294002 (a pan-PI3K inhibitor) or IC87114 (a p110δ-selective inhibitor). Both drugs were used at 10 µM for 10 min (*n*≥6 independent experiments). *p<0.01. (B) E13.5 DRG explants were cultured for 24 h on 20 µg/ml laminin and treated with Sema3A (0.3 µg/ml) for 30 min. Increased growth cone collapse in response to Sema3A (0.3 µg/ml) in p110δ KI DRGs (right). Each data point represents the %±SEM (*n*≥3 independent experiments). In each experiment at least 80 growth cones were counted. *p<0.02. (C) Example of DRG explants in each group, which have been stained for βIII-tubulin (*red*) and phalloidin (*green*). Scale bar, 20 µm.

**Figure 3:**
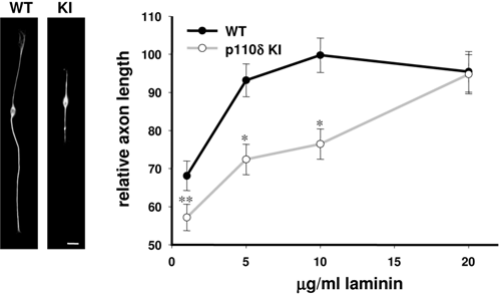
Reduced outgrowth of p110δ KI DRG neuron under limiting substrate conditions. *Left panel*, example of E13.5 DRG neurons derived from WT and p110δ KI mice, cultured on 10 µg/ml laminin for 24 h. *Right panel,* relative axonal length in p110δ KI and WT DRG neurons at 1, 5, 10 and 20 µg/ml laminin (right). Length was expressed relative to that of WT DRG neurons cultured in the presence of 10 µg/ml laminin. Each point represents the mean of at least 3 experiments±SEM, each experiment was carried out in duplicate. *n* = 60 neurons in each treatment. ^*^p<0.01.

We next assessed the responsiveness of neurons to PI3K inhibition using the pan-PI3K inhibitor LY294002 [1], which induces growth cone collapse in sensory neurons [16], [17]. Our analyses showed that this response was significantly greater in p110δ KI than in WT DRG neurons (Figure 2A). This indicates that DRG neurons with inactive p110δ are more sensitive to global PI3K inhibition, and also provides evidence that the remaining PI3K isoforms could not compensate for the loss of p110δ activity. IC87114, a p110δ-selective small molecule inhibitor [18], also induced growth cone collapse in WT DRG neurons, but had little effect on p110δ KI DRG neurons (Figure 2A), indirectly confirming the selectivity of this compound. Responsiveness of DRG neurons to physiological stimuli that utilize PI3K/Akt signaling was also assessed. Growth cone collapse induced by the axon guidance molecule Sema3A, known to decrease PI3K signaling [17], [19], was 50% higher in p110δ KI DRG neurons at low concentration of Sema3A (Figure 2B, C). Integrin activation through the substrate laminin is known to activate PI3K signaling in the growth cone [20]. Laminin-mediated axonal elongation in p110δ KI DRG neurons was reduced by almost 30% at lower (10 µg/ml) but not at higher (20 µg/ml) concentrations of laminin (Figure 3). Taken together, these observations uncover a p110δ PI3K signaling pathway, important for the maintenance of optimal axonal outgrowth in an inhibitory environment and under lower substratum availability. Genetic or pharmacological inhibition of p110δ in cultured DRG neurons did not alter apoptosis (as measured by apoptotic nuclei; data not shown), indicating that this pathway is independent of PI3K-mediated survival responses.

### Reduced axonal regeneration in the injured sciatic nerve of mice with inactive p110δ

**Figure 4:**
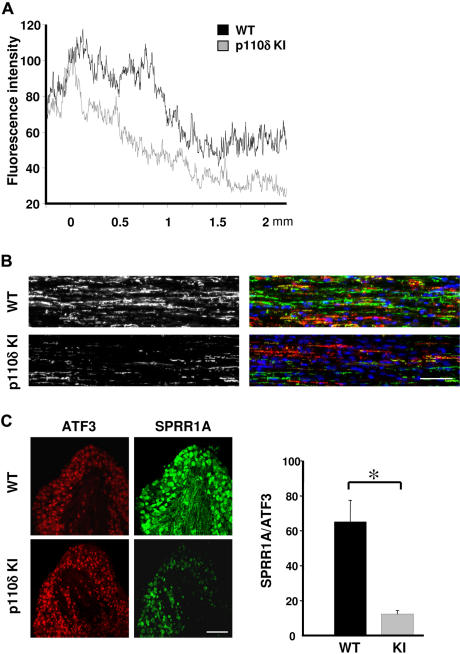
Reduced axonal regeneration in the injured sciatic nerve of p110δ KI mice. 3 days post-injury, the sciatic nerves of WT and p110δ KI mice were fixed and cryo-sectioned. (A) Average relative fluorescence intensity profile of anti-βIII-tubulin labeling across a one-pixel line along the entire nerve segment, following cropping of the micrographs to a fixed pixel segment. (B) High-power micrographs of the sciatic nerve segments 2 mm distal to the injury, labeled with anti-βIII-tubulin (left panels and green in right panels), anti-F4/80 (*red*) and Hoechst (*blue*). Scale bar, 50 µm. (C) At 7 days post injury, L4 DRGs of WT and p110δ KI mice were fixed, vibratome-sectioned, and co-labeled with ATF3 *(red)* and SPRR1A *(green)*. Percentage of SPRR1A and ATF3 co-labeling over ATF3-only positive DRG neurons in WT and p110δ KI mice. Data are from 3 WT and 4 p110δ KI mice, and are presented as mean±SEM. *p<0.05. Scale bar, 50 µm.

Given that the absence of p110δ activity limits axonal outgrowth in embryonic neurons, we assessed the axonal growth potential of adult p110δ KI neurons. Adult peripheral nerve axons are capable of functional regeneration in mammals, with a crush injury of the sciatic nerve being a well-established injury model to study axonal growth [21]. On day 3, extension of regenerating neuronal fibers 2 mm distal from the injury site was reduced in p110δ KI mice (Figure 4A,B), without any differences in the presence of cytoskeletal breakdown products 4 mm distally from the site of injury (data not shown), indicating that loss of p110δ activity does not alter normal axonal degeneration. p110δ activity has been shown to be essential for CSF-1-driven *in vitro* chemotaxis of macrophages [22], [23], which might affect axonal regeneration due to impaired inflammatory response at the injury site in p110δ KI mice. No changes in recruitment of macrophages (stained by F4/80) into the injured nerve were detected (Figure 4B). Next, we compared the capacity of DRG soma to upregulate growth-associated SPRR1A (small proline-rich repeat protein 1A) during regeneration. SPRR1A has been shown to peak 1–2 weeks following sciatic nerve injury in adult mice and its depletion reduces axonal outgrowth *in vitro* [24]. We co-stained for ATF3 (Activating Transcription Factor 3), which is produced *de novo* in sensory neurons following sciatic nerve injury and is widely used as a marker for nerve injury [25]. No significant difference was observed in the percent of ATF3-positive neurons in injured DRGs obtained from WT and p110δ KI mice in L4 DRGs 7 days after nerve injury, demonstrating that the sciatic nerve injury was equivalent in the two groups (data not shown). However, the regeneration marker SPRR1A was significantly reduced in L4 DRG soma of p110δ KI mice (Figure 4C), indicating that loss of p110δ function impairs regenerative capacity of adult sensory neurons.

### Inhibition of axonal regeneration correlates with impaired functional recovery

**Figure 5:**
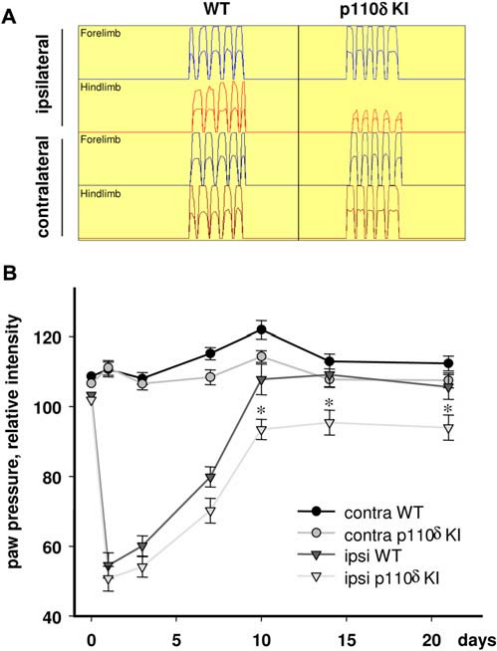
Impaired functional recovery in p110δ KI mice following sciatic nerve crush injury. Paw pressure intensity during continuous locomotion was assessed using the CatWalk quantitative gait analysis system. Recovery in locomotion was analyzed on days 1, 3, 7, 10, 14 and 21 post-injury. (A) Intensity profiles of 5 steps during a single run from each paw, 7 days post-injury. Each intensity profile shows two traces, a higher trace of the maximal relative intensity and a lower trace of the average relative intensity. (B) Reduced relative paw pressure intensity for each paw during recovery in p110δ KI mice compared to WT mice. Data presented is the mean±SEM of ≥6 animals. p<0.001, 2-way repeated measures ANOVA.

We next assessed whether the anatomical evidence for p110δ-dependency for optimal axonal regeneration correlated with functional recovery using an automated quantitative gait analysis system, the CatWalk, to assess recovery of locomotion following nerve injury on days 1, 3, 7, 10, 14 and 21 [26]. Prior to injury, gait analysis assessed by the CatWalk on a number of locomotor parameters revealed no differences between WT and p110δ KI mice (Figure 5A, B, S4). In contrast, following unilateral injury to the sciatic nerve, p110δ KI mice showed a significant decrease in the recovery of the ability to bear weight on the injured paw (Figure 5A, B). Whilst both WT and p110δ KI mice display functional recovery during the first 10 days post-lesion, the relative paw pressure intensity in p110δ KI mice was significantly lower in comparison to WT mice (Figure 5B; p<0,001, 2-way repeated measured ANOVA). These differences were most apparent at later time-points (day 10 and day 14 post-lesion; Figure 5B; p<0.05, Tukey test). These results indicate that following sciatic nerve injury, the lack of functional p110δ led to a decreased ability of the axons to undergo regenerative growth, which in turn led to decreased functional recovery.

### Reduced Akt and increased RhoA/PTEN signaling in neurons with inactive p110δ

**Figure 6:**
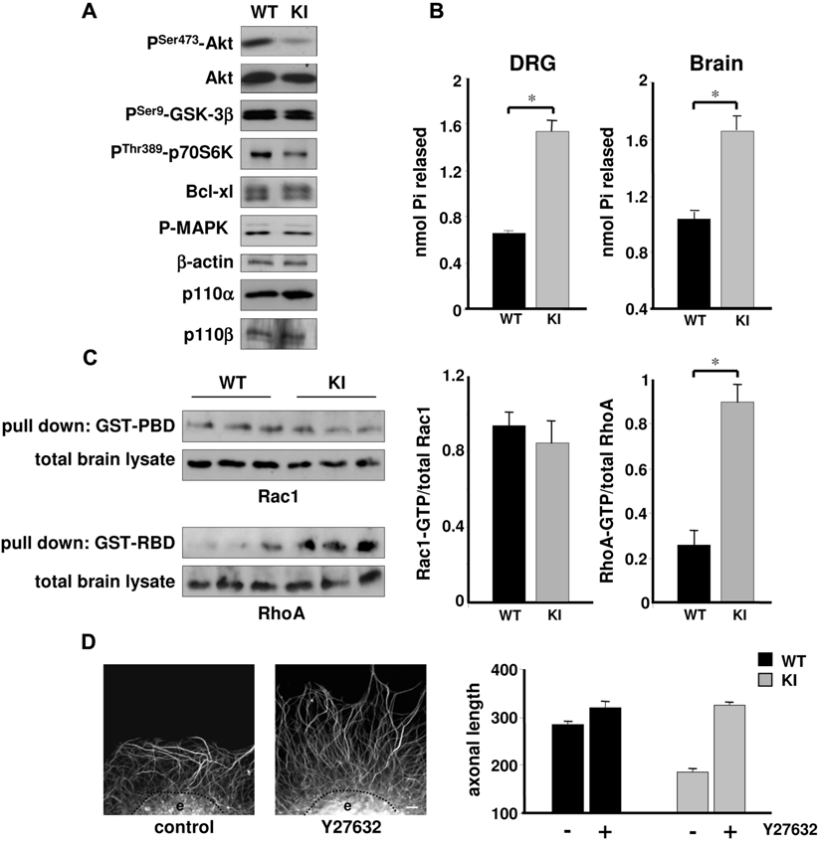
Reduced Akt and increased RhoA/PTEN signaling in DRG neurons with inactive p110δ. (Α) E13.5 DRG neurons of WT and p110δ KI mice were cultured for 24 h before processing for Western blot analysis using the indicated antibodies. (B) Inactivation of p110δ in p110δ KI mice leads to an increase in lipid phosphatase activity in PTEN immunoprecipitates from homogenates of brain *(left panel)* or DRGs *(right panel)* from WT and p110δ KI mice. Graphs show the lipid phosphatase activity in 3 independent experiments, measured in triplicates. p<0.001. (C) Inactivation of p110δ does not affect Rac, but increases RhoA activity. *Left panel,* brain lysate from WT and p110δ KI mice was subjected to pull-down with GST-PBD or GST-RBD, followed by SDS-PAGE and immunoblotting using antibodies to Rac1 or RhoA. *Right panel,* graphs represent the mean±SEM of Rac1-GTP (left) or RhoA-GTP loading of 2 experiments, each performed in triplicate. (D) Inhibition of ROCK rescues the effects of p110δ inactivation on axonal elongation at 10 µg/ml laminin. *Left panels,* example of an E13.5 DRG explant (e) of p110δ KI mice cultured for 24 h in the absence (control) or presence of Y27632 (10 µM). Scale bar, 20 µm. *Right panels,* length of axons extending from WT and p110δ KI DRG explants, in the absence (−) or presence (+) of Y27632 (10 µM). Each data point represents the mean±SEM of 3 independent experiments.

We next investigated the effect of p110δ inactivation on signaling in neurons. PI3K signaling drives many aspects of neuronal morphology through the coordinated phosphorylation of proteins that regulate cytoskeletal dynamics, protein synthesis, and transcriptional activity. Akt is an important effector through which PI3K controls axon elongation and morphological responses induced by neurotrophins [27], [28]. A substantial decrease in activatory Akt phosphorylation was observed in p110δ KI DRG neurons cultured in the presence of NGF (Figure 6A). In contrast, phosphorylation of GSK-3β, an effector of the PI3K/Akt pathway in many cell types [29] and a crucial determinant of axonal growth and guidance [27], [30], [31], was not affected (Figure 6A). This lack of inactivation of GSK-3β may allow the observed normal neurite outgrowth under non-limiting conditions in p110δ KI mice (Figure 3) and suggests the presence of alternative, p110δ-independent regulatory pathways for GSK-3β. Another member of the signaling pathway downstream of PI3K activation is mTor and its effector p70S6K, which controls the initiation of protein synthesis [1]. DRG neurons from p110δ KI mice showed a substantial decrease in p70S6K phosphorylation, whilst levels of MAPK phosphorylation and Bcl-xl protein were not affected (Figure 6A). These results indicate that p110δ PI3K activity exerts control over the Akt/p70S6K pathway, which is not compensated for by p110α or p110β.

p110δ has recently been demonstrated to inhibit the activity of the tumor suppressor PTEN through a pathway involving RhoA [23]. Similary, in p110δ KI DRGs and brain homogenates, PTEN lipid phosphatase activity was constitutively elevated (Figure 6B). In addition, GTP-loading of RhoA, but not Rac, was significantly increased in p110δ KI brain extracts (Figure 6C). These observation is in line with the idea that p110δ can suppress cellular RhoA but not Rac activity under basal conditions [23]. RhoA is a critical mediator of the inhibitory effect of several axon guidance molecules and myelin associated inhibitors [32], [33]. Inhibition of RhoA, or its downstream effector ROCK, is able to restore outgrowth in an inhibitory environment provided by myelin or myelin associated inhibitors [32], [33]. In order to test the significance of increased RhoA function for p110δ signaling during axonal elongation, we inhibited ROCK using the small molecule inhibitor Y27632 [34]–[36]. As expected, axons extending from p110δ KI DRG explants were significantly shorter than WT axons (Figure 6D). However, treatment with Y27632 restored axonal length in p110δ KI neurons to the lengths seen in WT neurons (Figure 6D). These data are consistent with a model whereby inactivation of p110δ leads to increases in ROCK activity as a consequence of higher levels of active RhoA. Previous work has indicated RhoA/ROCK signaling in the control of PTEN [37], thus raising the possibility that PTEN activity in neurons functions downstream of RhoA. Such deregulation of signaling by p110δ is consistent with the phenotypes observed in this study [17].

### Conclusion

The major finding of this study is the identification of a function of the p110δ PI3K in controlling effective axonal elongation under less favorable conditions and during insult to the nervous system. This is consistent with the idea that although p110δ is similar to p110α and p110β in terms of structure and substrate specificity, it is restricted in its expression and function (Figure 1; [7], [11], [38]). It remains to be determinded if the p110α and p110β isoforms of PI3K play similar roles in neurons. Homozygous inactivation of p110α or p110β leads to embryonic lethality [8], [9], [15], precluding investigation in this area until conditional p110α/p110β mutant mice will become available. p110δ is considered to be an interesting therapeutic target in inflammation and auto-immunity [12], [14], [39], and the development of small molecule inhibitors against p110δ is in progress [18]. The data reported here suggest that p110δ inhibitors may not have adverse effects on the steady-state functioning or the development of the nervous system. Nonetheless, caution should be exercised given that these compounds may have undesirable effects under conditions of nerve injury or ongoing neurological degeneration.

## Materials and Methods

### Mice

Gene targeting to create the p110α*^lz^* mice and p110δ*^lz^* mice has been described elsewhere [14], [15]. All mice were littermates and backcrossed for 10 generations onto the C57B16/J strain. Animals were maintained in individually-ventilated cages on a 12 h light-dark cycle, with free access to food and water. All experiments were undertaken in accordance with the UK Animals (Scientific Procedures) Act 1986.

### Embryo and tissue preparation

p110α*^lz^* and p110δ*^lz^* mice were transferred into ice-cold PBS, and fixed for 1 h on ice in fixing buffer (4% paraformaldehyde/0.2% glutaraldehyde/2 mM MgCl_2_/5 mM EGTA/0.02% NP40). Embryos were then washed 3 times in washing buffer (PBS/2 mM MgCl_2_/0.01% sodium deoxycholate/0.02% NP40/5 mM EGTA) and post-fixed in 4% paraformaldehyde for 1 h. β-galactosidase expression was visualized by incubation in X-gal developing buffer (PBS/5 mM K_3_Fe(CN)_6_/5 mM K_4_(CN)_6_/2 mM MgCl_2_/0.01% sodium deoxycholate/0.02% NP40, 1 mg/ml X-gal) overnight at room temperature. Embryos were then washed in PBS, post-fixed in 4% paraformaldehyde for 1 h, and washed twice in distilled water before dehydration though a series of 70% ethanol, 95% ethanol, and 100% ethanol. Following incubation in methylsalicylate for 1 h, embryos were rehydrated in 100% ethanol, 95% ethanol, 70% ethanol, and 30% ethanol and finally washed in PBS before being embedded in 10% gelatin and vibratome-sectioned at 50 µm.

### Sciatic nerve crush

For *in vivo* regeneration studies, 6 to 8 week-old male mice were used. The mice were assessed before surgery to establish baseline-walking patterns. Animals were anesthetized with a mixture of medetomidine (0.5 mg/kg) and ketamine (75 mg/kg), and the left sciatic nerve was exposed. A crush injury was performed 10 mm distal to the obturator tendon using forceps compression (10 sec) and the crush site labeled with lamp black. The muscle and skin layers were sutured and animals were allowed to recover in the cage post-operatively.

### The CatWalk

The CatWalk gait analysis system was used to assess functional recovery of locomotion following sciatic nerve crush injury [26]. The animal traversed a meter long walkway with a glass floor and 2 perspex walls spaced 8 cm apart, housed in a darkened room. Light from 2 encased white fluorescent tubes entered the glass floor through the distal edge of the glass, and was totally internally reflected. Light scatters only where a paw contacts the glass, illuminating the area of paw contact. This reflected light was captured using videocamera (Sentech 705, 8.5 mm, f = 1.4, variable focus and variable iris) equipped with a wide-angle objective and a frame grabber (Matrix Vision SG-board) connected to a PC running the CatWalk 500 software for capture and analysis [26]. Each mouse ran across the CatWalk one day before surgery to establish baseline locomotor parameters. Following surgery, animals ran the Catwalk on days 1, 3, 7, 10, 14 and 21. The program was set to capture the paw prints from the middle section of the run. At least 2 runs per animal were performed on each day. Data was analyzed by labeling all areas containing one or more pixels above a certain analysis threshold. In a second interactive pass, these areas were assigned to each of the paws (left and right fore and hind paws: LF, RF, LH, RH). Data generated from the program was exported to Excel, yielding several parameters including average area and intensity for each paw, the regularity index, and duration of swing and stance phase. Statistical significance was evaluated using two-way repeated measures ANOVA and Tukey *post-hoc* comparisons (Sigma Stat 3.0.1, SPSS Inc.).

### Morris water maze

Mice for behavioral testing were housed in groups of 2 to 4 in individually-ventilated cages, with food and water *ad libitum* and maintained on a 12 h light-dark cycle. Mice tested were males between the ages of 2 and 6 months. The Morris water maze protocol has been described previously [40]. Briefly, the mice were trained in a 1.5 m pool with a 10 cm platform. The water was maintained at 24–27°C, and made opaque with non-toxic white paint. The animals received 12 training trials per day, in blocks of 4, with 1 h in between each block, and a 90 sec maximum swim time. On days 3 and 5, a probe trial was given, in which the platform was removed and the animal was allowed to swim for 90 sec before being removed from the pool. The movement of the animals whilst in the pool was videotaped and recorded by a computer tracking system (HVS Image, Hampton, UK). The behavioral data were analyzed with the ‘HVS water program’ and Sigmastat (SYSTAT Software, SSPS Science Inc).

### Immunohistochemistry

For stainings of DRG neurons *in situ*, E13.5 WT and p110δ KI mice were paraformaldehyde fixed, embedded in 20% gelatin and vibratome-sectioned at 80 µm. Anti-P-p70S6K (Cell Signaling Technology) was applied at 1∶300 overnight at 4°C. Following extensive washes, bound antibodies were detected using Alexa conjugated secondary antibodies (1∶1000). Sections of WT and p110δ KI mice were analyzed by confocal microscopy at the same scanning settings for direct intensity comparison (optical slice size 2 µm, Zeiss LSM 5 META confocal laser scanning microscope). For assessment of axon regeneration in the peripheral nerve, mice were deeply anaesthetized with pentobarbitone (80 mg/kg, i.p.) 3 days after nerve crush and transcardially perfused with 10 ml saline followed by 50 ml paraformaldehyde (4% in 0.1 M phosphate buffer). The entire left and right sciatic nerves were removed, post-fixed in 4% paraformaldehyde for 2 h, transferred to 20% sucrose overnight, mounted in OCT (BDH, UK), and cryosectioned longitudinally for subsequent immunohistochemistry using anti-βIII-tubulin antibody (1∶800, Covance), anti-F4/80 (Serotec) and the nuclear dye Hoechst (Sigma). For assessment of regeneration-associated markers in the cell bodies, 7 days after the sciatic nerve crush, mice were sacrificed (as above). DRGs from lumbar segments 4 and 5 were dissected, stored in 4% paraformaldehyde at 4°C for 2 h, washed once in PBS and embedded in 10% gelatin, followed by cutting of 20 µm sections on ice using a vibratome (Leica, Speed 3-Frequency 7). Sections were transferred to PBS+1% sodium azide, and anti-SPRR1A antibody (kindly provided by S. Strittmatter, Yale University) was applied at 1:7000 in PBS/0.2% Triton-X100 and incubated overnight at room temperature, under mild agitation. Sections were washed extensively, and bound antibody was detected using biotinylated horse anti-mouse secondary antibody (Jackson ImmunoResearch; 1∶400, 90 min), ABC reagent (Vector Labs; 1∶250, 30 min), biotinyl tyramide (PerkinElmer Life Sciences; 1∶75, 10 min) and extra-avidin FITC (Sigma; 1:500, 2 h). Sections were then incubated with rabbit anti-ATF3 (1∶400, Santa Cruz), overnight at 4°C. After extensive washes, sections were incubated with donkey anti-rabbit TRITC (1∶200, Jackson). All sections were mounted on slides in Vectashield fluorescent mounting medium (Vector Laboratories Ltd., UK).

### Neuronal cultures

Age-matched WT and p110δ KI embryos were isolated at the appropriate age, and transferred into ice-cold DMEM. DRGs were dissected from E13.5 mouse embryos and extramesenchymal tissue was removed using a sharpened 0.2 mm tungsten wire. DRGs were then plated on glass coverslips previously coated with poly-L-lysine and laminin (both at 20 µg/ml; Sigma). For primary neuronal cultures, DRGs were incubated in trypsin (1 mg/ml, diluted in HBSS) for 10 min at 37°C, and dissociated using a fire-polished Pasteur pipette. Neurons were either cultured at low density (50 cells/mm^2^) for neurite outgrowth assays on glass coverslips coated as described above, or in laminin-coated 6-well dishes (150 cells/mm^2^) for biochemical analysis. Explants and primary DRG neurons were incubated at 37°C/5% CO_2_ for 24 h in DMEM/10% FCS/Pen/Strep supplemented with 20 ng/ml NGF (Promega). Pharmacological inhibitors were used as described; LY294002 was purchased from Calbiochem. The collapse assay using purified Sema3A-Fc or PI3K inhibitors was performed as previously described [17].

### Immunocytochemistry

Neuronal cultures were treated as indicated and paraformaldehyde-fixed (4% paraformaldehyde/PBS/10% sucrose) for 30 min before permeabilization for 5 min with PBS/1% Triton ×100. Neurons were then labeled with Phalloidin-Alexa488 (1∶50 in PBST) and anti-βIII-tubulin antibody (1∶800; Covance). For each experiment, the collapsed growth cones were counted and represented as a % ratio. Each experiment was performed a minimum of 4 times, and the average % collapsed was determined. Standard errors of the mean were determined as the (standard deviation/square root (number of experiments)). For determination of neurite length, neurons were labeled with the anti-βIII-tubulin antibody, and the KS300 program (Zeiss) was used to measure neurite length in each treatment.

### SDS-PAGE and Western blotting

Dissociated neurons were washed twice with ice-cold PBS lysed for 30 min in ice-cold lysis buffer (10 mM Tris.HCl pH 7.4, 250 mM sucrose, 10 mM MgCl_2_, 0.5% NP40, complete protease inhibitor cocktail (Roche), 2 mM sodium orthovanadate, 0.1 mM DTT, 25 mM NaF). All cell lysates was adjusted to equal concentrations, and 20 µg protein was separated by SDS-PAGE and blotted onto nitrocellulose (Hybond ECL, Amersham Biosciences). Primary antibodies were applied for 1 h followed by 3 washes in TBST. Antibody sources were as follows: anti-P-Akt, anti-P-p70S6K, anti-P-MAPK, anti-Bclxl and anti-P-GSK-3β (Cell Signaling Technology), anti-actin (Roche), anti-p110β (sc-602; Santa Cruz). Antibodies to p110α or p110δ were generated in-house [22]. Bound antibody was detected using HRP-conjugated secondary antibody (Vector Labs) diluted in blocking milk, which was applied for 1 h. After extensive washes in TBST, blots were developed on MXB film (Kodak) using an ECL (Amersham Biosciences) detection system.

### GTPase and PTEN lipid phosphatase activity assays

The Rac or RhoA activation assays were performed using GST-PBD (p21-binding domain of PAK) or GST-RBD (Rho binding domain of Rhotekin), respectively, as described [23]. In brief, brain tissue was lysed in Mg^2+^ lysis buffer (Upstate) and mixed with GST-PBD or with GST-RBD bound to glutathione-agarose and incubated for 1 h at 4°C. Bound protein was washed, and suspended in sample buffer. Proteins were then separated by SDS-PAGE, transferred to PVDF membranes and blotted with the indicated antibodies. PTEN lipid phosphatase activity was measured as previously described using malachite green reagent for the detection of phosphate release [23]. Similar results were also obtained using a PTEN activity ELISA kit (Echelon).

## Supporting Information

Figure S1 Expression of p110δ and other class IA PI3K isoforms in the brain. Coronal sections of the brain of (A) p110δ lz and (B) WT adult mice reveal restricted expression of p110δ/LacZ in several brain regions, including the cortex (Cx), hippocampus (H) and thalamus (Th). Sections were counterstained with nuclear fast red. Scale bar, 1 mm. (C) p110δ expression in different brain areas as assessed by X-gal staining of adult lacZ (β-Gal) reporter mice. (D) Expression of PI3K isoforms and the CD45 pan-leukocyte marker in lysates of different brain regions and thymus of adult WT mice. CD45 was found to be expressed in thymus and not in the brain, indicating that X-gal signals do not derive from resident leukocytes in the brain.
(1.37 MB TIF)

Figure S2 Expression of class IA PI3K proteins in the hippocampus of p110δ KI mice. Tissue extracts from the hippocampus from adult WT and p110δ KI mice were immunoblotted with PI3K isoform-specific antibodies as indicated. Anti-β-actin staining was used as internal control for equal protein loading.
(0.15 MB TIF)

Figure S3 Normal spatial memory development of p110δ KI mice in the Morris water maze. (A) WT and p110δ KI mice were trained with 12 trials per day in blocks of 4 trials. The time to reach the hidden platform is shown; there was no difference between the genotypes. (B) After training, day 3 and 5 probe trials were performed to assess selective searching in the quadrant where the platform used to be (TQ). Both genotypes searched selectively indicating normal spatial memory in p110δ KI mice. (C) The ‘platform crossings’ during the probe trials showed the same accuracy in WT and p110δ KI mice. Each data point represents the mean+SEM (n = 11 mice/group). During the probe trials the swim speeds did not differ between the genotypes (data not shown).
(1.70 MB TIF)

Figure S4 p110δ KI mice display normal locomotor parameters prior to injury. WT and p110δ KI mice were assessed for 6 locomotor parameters using the CatWalk quantitative gait analysis system to obtain baseline values. (A) The Regularity Index, an index that quantifies the % of steps assigned to one of 6 normal step sequences [26], is equivalent between WT and p110δ KI mice. (B) The base of support (measured in arbitrary units) represents the width between the two hind paws and indication of the stability of posture during locomotion. The base of support does not differ between WT and p110δ KI mice. (C, D) For each hind paw, the average area of contact and the average intensity of light reflected at each point of contact (which is indicative of the pressure applied by the paw upon the glass surface) are equivalent between WT and p110δ KI mice. Both average area and average intensity are measured in arbitrary units. (E, F) The stance phase is timed while the paw is placed upon the glass and the swing phase is timed between paw placements. The duration of swing and stance phases (in sec) between the two groups do not differ. In each evaluation, every data point represents the mean+SEM (n = 6 mice/group). p>0.1 in all parameters.
(0.47 MB TIF)
