# Supplementary figures and images for: Control of Axonal Growth and Regeneration of Sensory Neurons by the p110δ PI 3-Kinase
Source: PLoS One. 2007 Sep 12;2(9):e869. doi: 10.1371/journal.pone.0000869 (PMC1959241; doi:10.1371/journal.pone.0000869)

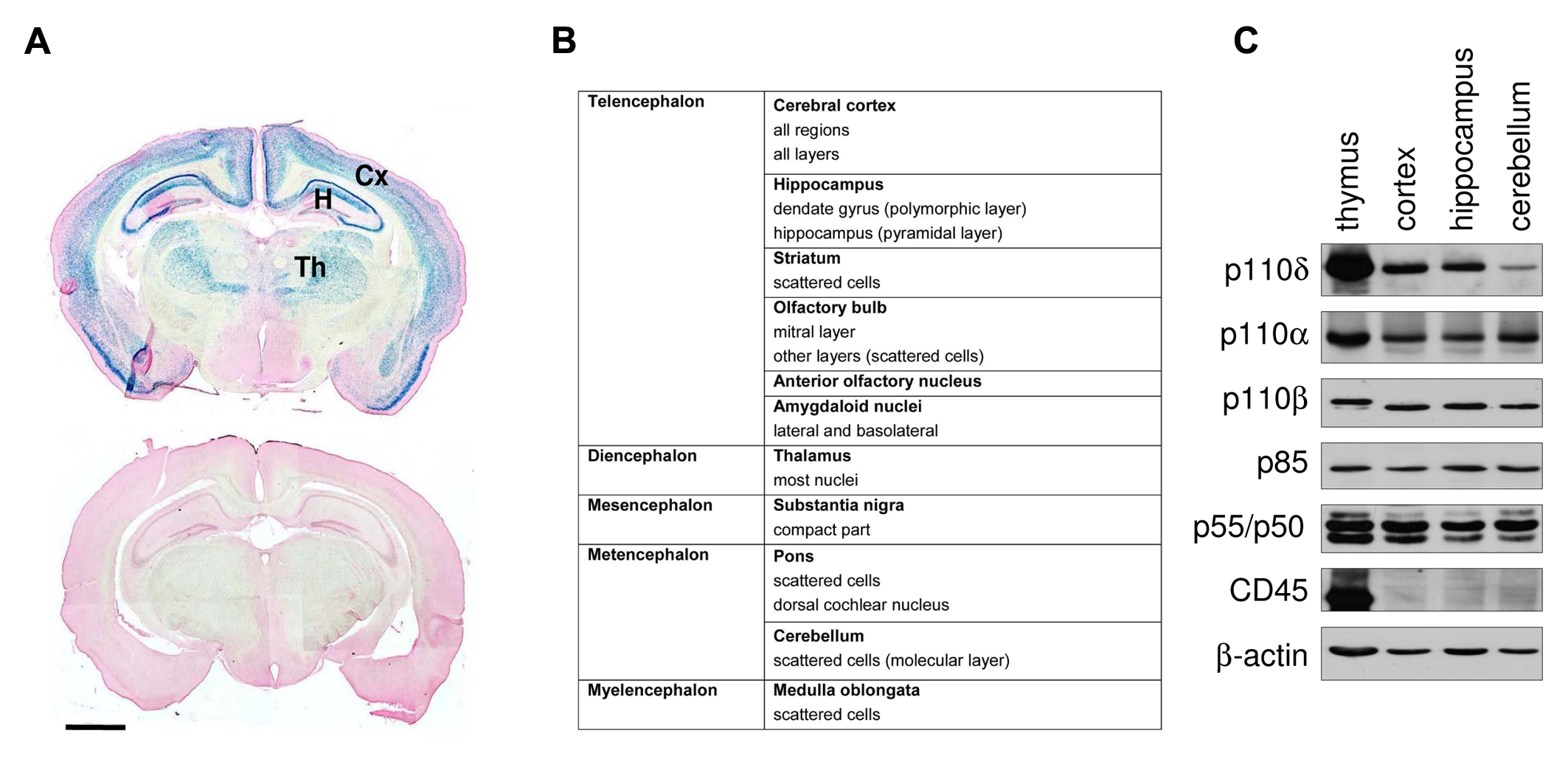

Supplement: Figure S1 — Expression of p110δ and other class IA PI3K isoforms in the brain. Coronal sections of the brain of (A) p110δ lz and (B) WT adult mice reveal restricted expression of p110δ/LacZ in several brain regions, including the cortex (Cx), hippocampus (H) and thalamus (Th). Sections were counterstained with nuclear fast red. Scale bar, 1 mm. (C) p110δ expression in different brain areas as assessed by X-gal staining of adult lacZ (β-Gal) reporter mice. (D) Expression of PI3K isoforms and the CD45 pan-leukocyte marker in lysates of different brain regions and thymus of adult WT mice. CD45 was found to be expressed in thymus and not in the brain, indicating that X-gal signals do not derive from resident leukocytes in the brain. (1.37 MB TIF) [file pone.0000869.s001.tif]

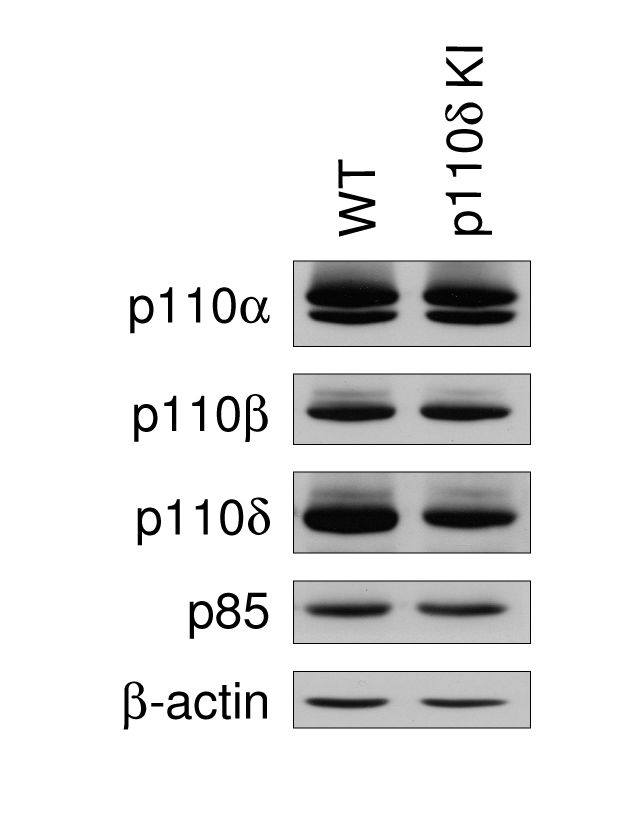

Supplement: Figure S2 — Expression of class IA PI3K proteins in the hippocampus of p110δ KI mice. Tissue extracts from the hippocampus from adult WT and p110δ KI mice were immunoblotted with PI3K isoform-specific antibodies as indicated. Anti-β-actin staining was used as internal control for equal protein loading. (0.15 MB TIF) [file pone.0000869.s002.tif]

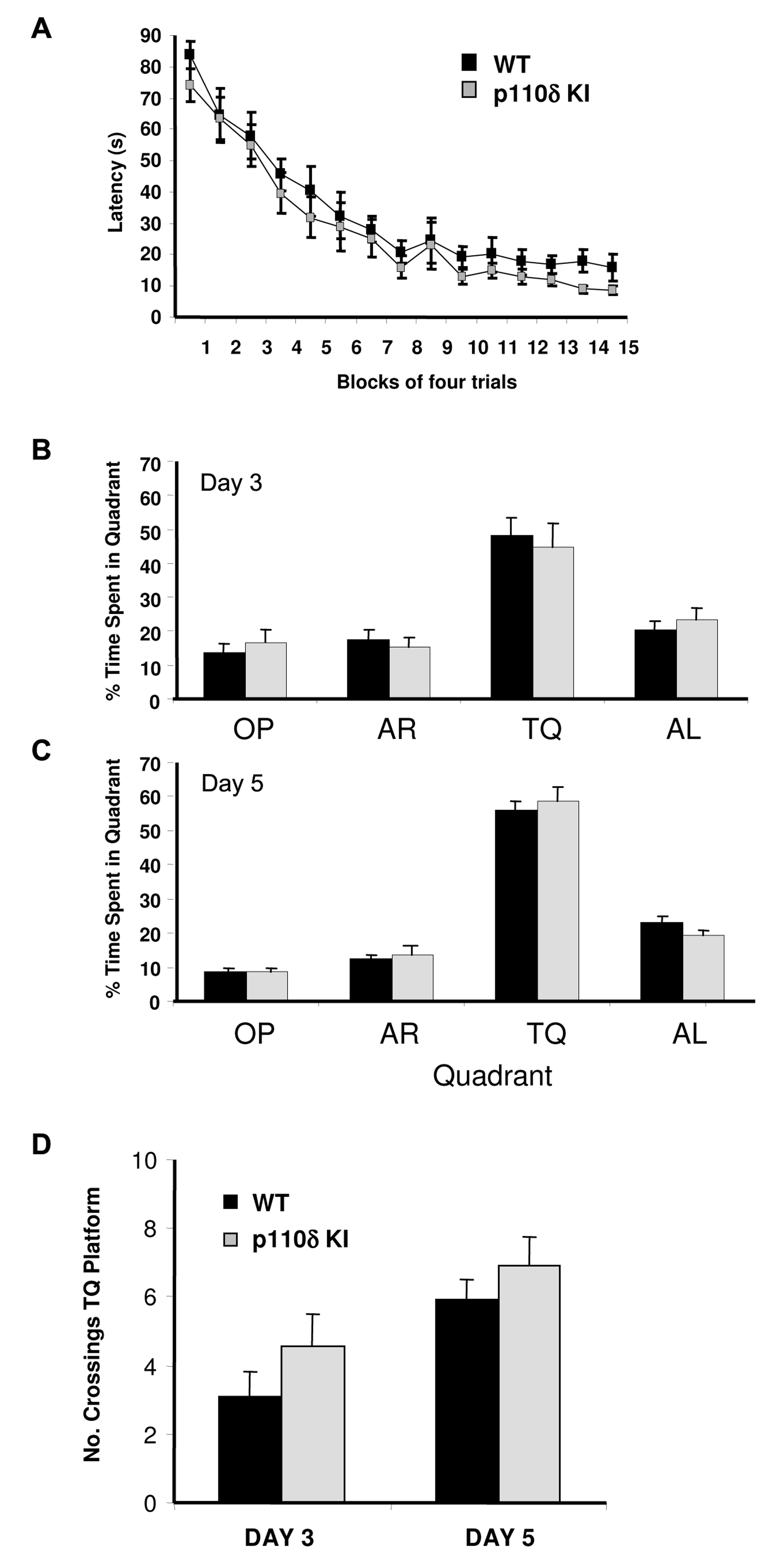

Supplement: Figure S3 — Normal spatial memory development of p110δ KI mice in the Morris water maze. (A) WT and p110δ KI mice were trained with 12 trials per day in blocks of 4 trials. The time to reach the hidden platform is shown; there was no difference between the genotypes. (B) After training, day 3 and 5 probe trials were performed to assess selective searching in the quadrant where the platform used to be (TQ). Both genotypes searched selectively indicating normal spatial memory in p110δ KI mice. (C) The ‘platform crossings’ during the probe trials showed the same accuracy in WT and p110δ KI mice. Each data point represents the mean+SEM (n = 11 mice/group). During the probe trials the swim speeds did not differ between the genotypes (data not shown). (1.70 MB TIF) [file pone.0000869.s003.tif]

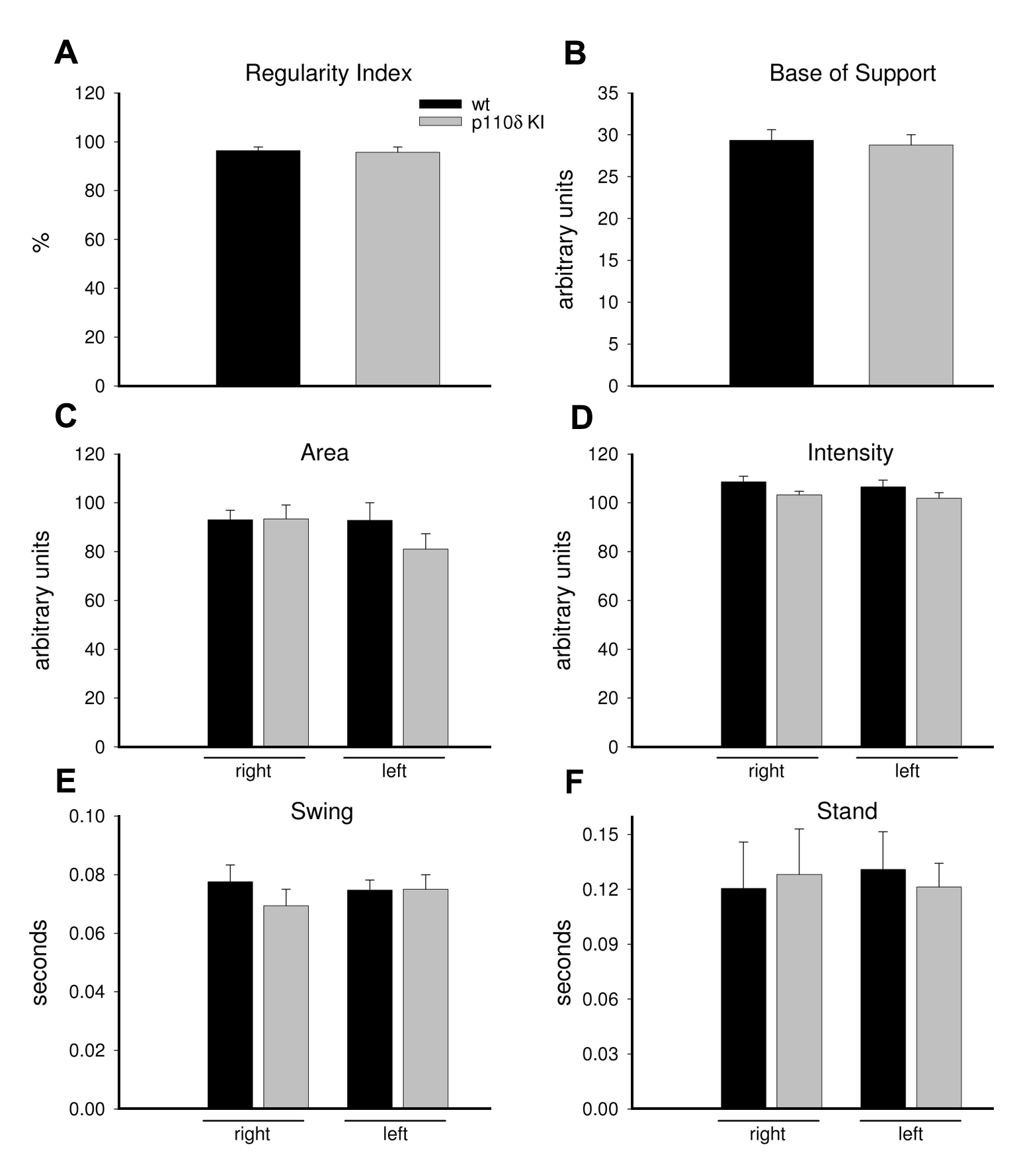

Supplement: Figure S4 — p110δ KI mice display normal locomotor parameters prior to injury. WT and p110δ KI mice were assessed for 6 locomotor parameters using the CatWalk quantitative gait analysis system to obtain baseline values. (A) The Regularity Index, an index that quantifies the % of steps assigned to one of 6 normal step sequences [26], is equivalent between WT and p110δ KI mice. (B) The base of support (measured in arbitrary units) represents the width between the two hind paws and indication of the stability of posture during locomotion. The base of support does not differ between WT and p110δ KI mice. (C, D) For each hind paw, the average area of contact and the average intensity of light reflected at each point of contact (which is indicative of the pressure applied by the paw upon the glass surface) are equivalent between WT and p110δ KI mice. Both average area and average intensity are measured in arbitrary units. (E, F) The stance phase is timed while the paw is placed upon the glass and the swing phase is timed between paw placements. The duration of swing and stance phases (in sec) between the two groups do not differ. In each evaluation, every data point represents the mean+SEM (n = 6 mice/group). p>0.1 in all parameters. (0.47 MB TIF) [file pone.0000869.s004.tif]
